# Successful implantation of an extravascular implantable cardioverter-defibrillator in a patient with moderate pectus excavatum: a case report

**DOI:** 10.1093/ehjcr/ytaf331

**Published:** 2025-07-17

**Authors:** Holden Eaton, Hesham Aggour, Julian O M Ormerod

**Affiliations:** University of Oxford Medical School, John Radcliffe Hospital, Headley Way, Headington, Oxford OX3 9DU, UK; Department of Cardiovascular Medicine, University of Oxford, Wellington Square, Oxford OX1 2JD, UK; Department of Cardiovascular Medicine, University of Oxford, Wellington Square, Oxford OX1 2JD, UK

**Keywords:** ICD, Pectus excavatum, EV-ICD, Ventricular fibrillation, Case report

## Abstract

**Background:**

Implantable cardioverter-defibrillators (ICDs) prevent sudden cardiac death due to ventricular arrhythmia. A novel extravascular ICD (EV-ICD) system provides improved functionality over previous transvenous (TV-ICD) and subcutaneous (S-ICD) alternatives, particularly in younger patients. This includes limited bradycardia pacing, anti-tachycardia pacing therapy, and lower energy defibrillation, all within a smaller device profile compared to the S-ICD. Due to the need for substernal lead placement, however, complex sternal anatomy is currently considered a relative contraindication to their use.

**Case summary:**

We present a 38-year-old male patient with pectus excavatum and a previous episode of ventricular fibrillation leading to out-of-hospital cardiac arrest. Initial implantation of a S-ICD was associated with repeated inappropriate shocks over 4 years, leading to multidisciplinary discussions with the patient regarding off-label use of an EV-ICD, as approved by Medtronic CRM. Explantation of the S-ICD and implantation of the EV-ICD were successful, with minimal technical changes required compared to standard surgical technique in normal sternal anatomy. At 6-month follow-up, there were no complications or inappropriate shocks.

**Discussion:**

The EV-ICD may be considered in patients with pectus excavatum, making this novel device available to a larger proportion of patients. Aside from careful consideration of instrument positioning during the procedure, this can be done with minimal changes to the standard surgical protocol.

Learning points

- Extravascular ICDs provide increased functionality over subcutaneous devices—including anti-tachycardia pacing, bradycardia pacing, and reduced defibrillation thresholds—whilst avoiding the intravascular space.
- Complex sternal anatomy is currently considered a relative contraindication to the EV-ICD, as this subgroup was not included in initial clinical trials.
- This case highlights the feasibility of EV-ICD use in a patient with pectus excavatum, with minimal change to surgical technique and therefore the potential for expanded use of this valuable therapeutic device.

## Introduction

Sudden cardiac death (SCD) remains a major challenge in western societies, accounting for 20% of all natural deaths despite diagnostic and therapeutic advancements in cardiovascular diseases.^1^ The implantable cardioverter-defibrillator (ICD) is an established therapy for the prevention of SCD secondary to ventricular arrhythmias (VAs).^2^ Transvenous ICDs (TV-ICDs), implanted in a similar manner to pacemakers (with leads in the heart), have been available since the 1980s^3^; however, the risk of infection/endocarditis and vascular complications associated with their use have encouraged the development of alternative devices.

The subcutaneous ICD (S-ICD) entered widespread clinical use in the 2010s and was the first ICD to be entirely outside the heart and great vessels. The lead is implanted superficial to the sternum, with the generator (‘can’) in the axilla. The suprasternal positioning leads to higher energy requirements for effective defibrillation; the battery and capacitor are therefore significantly larger than those in TV-ICDs, and battery life is shorter. The S-ICDs also cannot offer bradycardia pacing or anti-tachycardia pacing (ATP). The S-ICD has found a particular niche in younger patients without pacing indications, who may have many decades of ICD therapy and therefore a high lifetime risk of TV-ICD complications.

The extravascular ICD (EV-ICD) system was developed to address some of the drawbacks of the S-ICD (Summary *figure*). The novel lead is implanted deep to the sternum, in the anterior mediastinum. The lead is therefore closer to the heart, allowing for limited bradycardia pacing, ATP therapy, and lower energy defibrillation. The can is around half the size of the S-ICD with longer predicted battery life.

Contraindications to EV-ICD implantation predominantly relate to the lead position. Prior sternotomy is an absolute contraindication as the space is obliterated. Significant sternal abnormalities like pectus carinatum or excavatum are considered relative contraindications and an off-label use of the device.^4^ However, such chest wall structural deformities are common, affecting up to 0.4% of the population,^5^ and clinicians should be alert to the risk of denying this new device option inappropriately.

We present a case of a young male patient who underwent successful S-ICD extraction and EV-ICD implantation in the context of pectus excavatum.

## Summary figure

**Figure:**
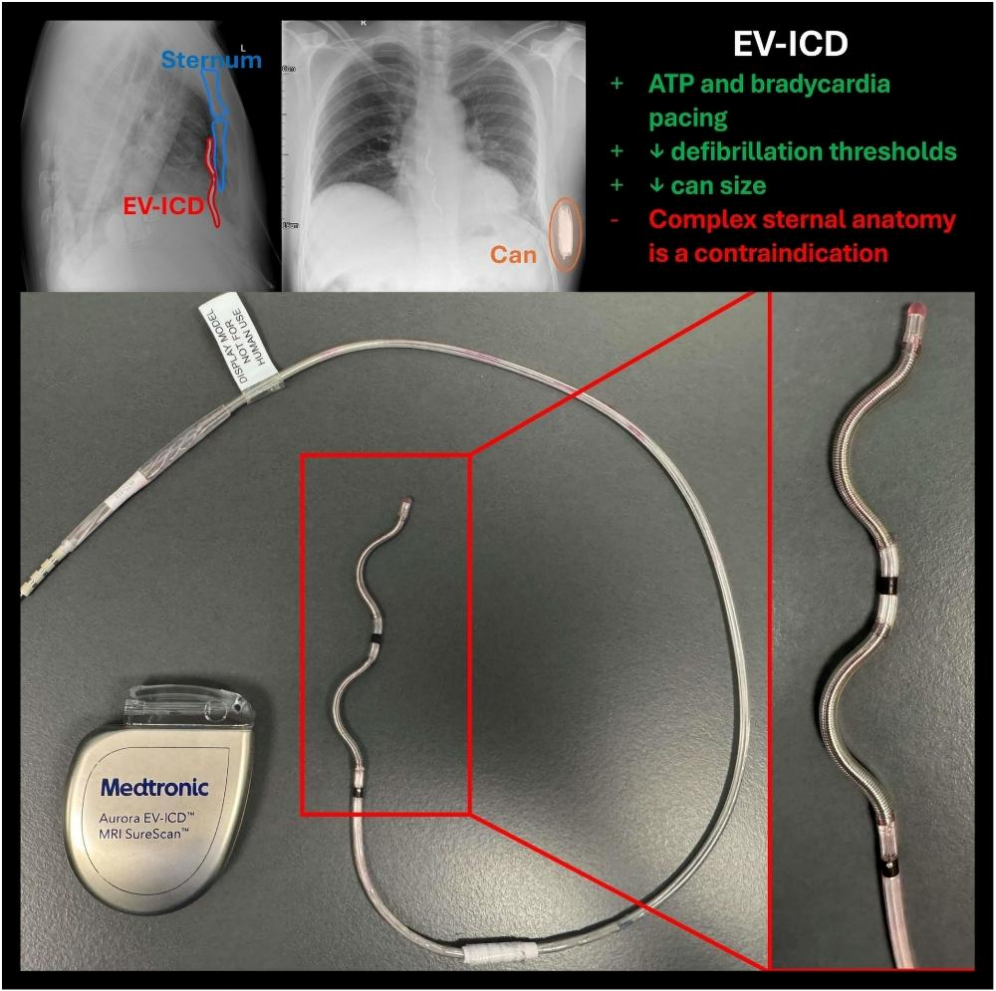
(*A*) Extravascular ICD lead in relation to the sternum in pectus excavatum (see antero-posterior and lateral chest X-ray views). (*B*) Dummy device (EV-ICD) used for patient education and pre-operative planning; of note is the reduced can size (64 × 51 × 13 mm) compared with prior SC-ICD (83.1 × 69.1 × 12.7 mm) and novel lead design.

## Case summary

**Figure 1:**
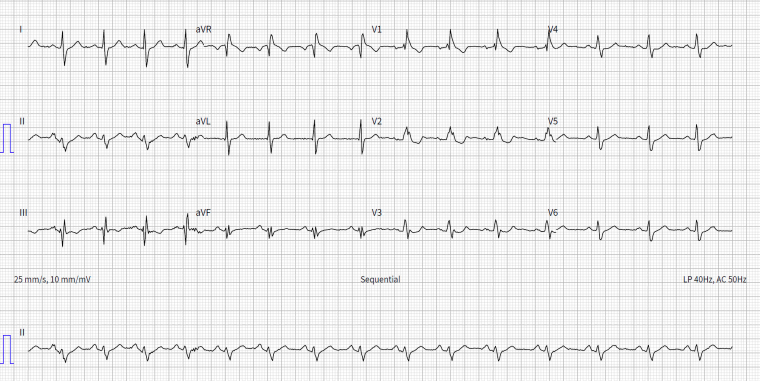
Resting 12-lead electrocardiogram—normal sinus rhythm and right bundle branch block with no evidence of conduction abnormalities or channelopathies.

A 38-year-old male patient presented in July 2017 following an out-of-hospital cardiac arrest due to ventricular fibrillation (VF). His resting 12-lead electrocardiogram showed normal sinus rhythm and right bundle branch block with no evidence of conduction abnormalities or channelopathies (*Figure 1*), and the ajmaline provocation test was negative. Both transthoracic echocardiogram and invasive coronary angiogram showed normal anatomy. He had no family history of SCD, and blood tests were unremarkable. The only notable finding was moderate pectus excavatum (Haller index = 2.8).^6^ A diagnosis of idiopathic VF was made. A Boston Scientific Emblem^™^ MRI S-ICD system was implanted 10 days later after favourable pre-implantation S-ICD screening.

Over the next 4 years, lead noise in the primary vector was detected during follow-up. Despite fluoroscopic surveillance and provocation testing ruling out lead fracture, he experienced two inappropriate shocks (IAS) due to T wave oversensing. Repeated screening revealed limited sensing vector options. The high emotional and physical burden associated with IAS caused the patient to seek alternative therapies to his current S-ICD. A multidisciplinary team review suggested using an EV-ICD to avoid the risks associated with TV-ICDs. The patient, having been informed of the relative contraindication of his pectus excavatum, was keen to proceed.

**Figure 2:**
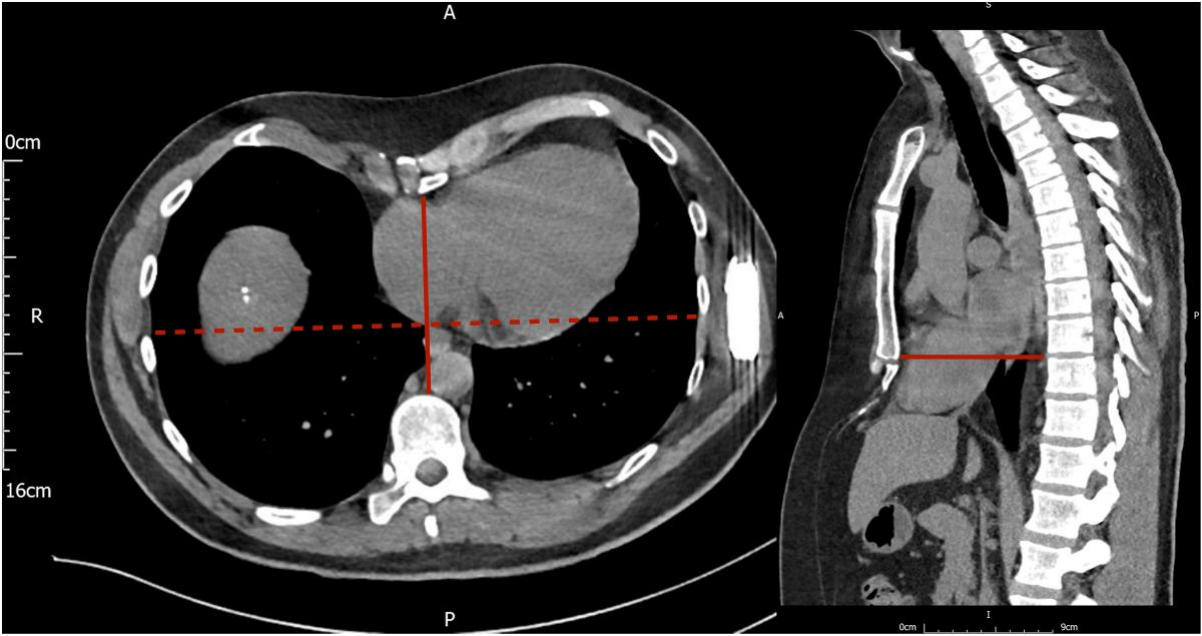
Pre-operative computed tomography scan—pre-operative computed tomography scan for surgical planning, demonstrating the degree of pectus excavatum; axial view (left) and sagittal view (right).

Pre-operative planning with a computed tomography (*Figure 2*) clarified the degree of pectus excavatum and anatomical landmarks, and the procedure was performed under a general anaesthetic. The S-ICD was first explanted without complication. Anatomical landmarks for the EV-ICD were marked, and then, an incision was made inferior to the xiphisternum to access the substernal space. Blunt dissection was performed to reach the pre-rectus plane, rectus muscle, and finally through the diaphragm. Particular care was taken to ensure access was made into the mediastinal rather than pericardial or pleural spaces. The Epsila EV^™^ sternal tunnelling tool was carefully advanced superiorly within a SafeSheath® peel-away sheath, with sequential orthogonal fluoroscopic views confirming the correct course (*Videos S1* and *S2*). On lateral fluoroscopic projections, the pectus caused distortion of the view, giving a misleading impression of the tunnelling tool. Particular care was therefore taken to angle the tunnelling tool away from the pericardium, by maintaining contact with the posterior sternum. The tunnelling path was then extended to the upper border of the cardiac silhouette. Once in place, the tunnelling tool was removed, and the lead was advanced via the peel-away sheath.

**Figure 3:**
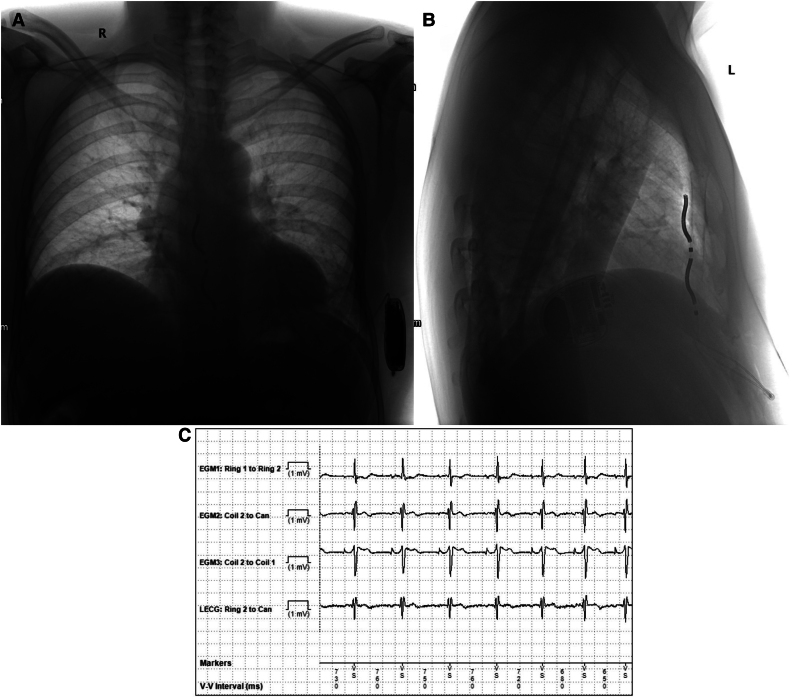
Post-procedure chest X-ray and extravascular implantable cardioverter-defibrillator electrogram—(*A*) antero-posterior chest X-ray after extravascular implantable cardioverter-defibrillator implantation. (*B*) Lateral chest X-ray highlighting position of extravascular implantable cardioverter-defibrillator lead in relation to sternum. (*C*) Extravascular implantable cardioverter-defibrillator intra-cardiac electrogram: R wave amplitude was 2.0 mV and a small p wave was visible but not detected by the device.

The remainder of the procedure followed standard implant protocol (*Video S3*). The lead parameters were satisfactory (*Figure 3*), and defibrillation threshold testing was successful at 30 J, giving an extra 10 J safety margin for programmed output. Post-operative chest X-ray confirmed appropriate lead position (*Figure 3*), and the patient was successfully discharged the following day. At 6-month follow-up, device interrogation showed stable lead parameters with no therapy delivered.

## Discussion

The European Society of Cardiology recommends ICD implantation in the management of idiopathic VF as a Class I indication.^2^ The S-ICDs have become a valuable alternative to TV-ICDs for the management of potentially fatal VAs, with lower rates of intravascular lead-related complications such as lead dislodgement, lead failure, cardiac perforation, and pneumothorax.^7^

The S-ICDs are, however, limited by their unique lead placement. Anti-tachycardia pacing is an established ICD therapy that can terminate ventricular tachycardia (VT) without painful shocks. The PAINFREE study demonstrated successful ATP termination of up to 80% of fast VT events in a broad ICD population.^8^ Although it has been demonstrated that S-ICDs can be safely used in patients with chest wall deformities,^9^ the greater distance between the lead and myocardium precludes the possibility of ATP and increases defibrillation thresholds. The S-ICDs are therefore associated with painful shocks for VT that could otherwise be avoided if ATP was possible.

The EV-ICDs, such as Medtronic’s Aurora EV-ICD^™^ system, offer solutions to both TV and SC limitations. The substernal lead positioning offers greater proximity to the heart than S-ICDs whilst avoiding intravascular lead-related complications (see Summary *figure*). This also allows for ATP therapy, more effective post-shock bradycardia pacing, and reduced defibrillation thresholds.^10^ In addition, a lower energy requirement allows for a smaller and therefore more comfortable can and potentially more streamlined generator replacements in the future.

The EV-ICDs are not without limitation, as accessing the substernal space may create additional risk to the pericardium, pleural, and abdominal space. The commonest major complications of EV-ICD use identified in the ‘PIVOTAL’ trial were lead dislodgement and post-operative device-pocket-wound infection (both also common to other ICD classes).^10^ Patients with significant chest wall abnormalities or past history of sternotomy were excluded from the trial^10^ as such factors might increase risk of injury to surrounding structures, and hence, EV-ICDs are not licensed in this group. There has, however, been at least one successful case of EV-ICD implantation in a patient following cardiac surgery reported in the literature.^11^

This case demonstrates the feasibility of safe EV-ICD implantation in patients with chest wall deformities. In addition to standard operating practice and close attention to surgical plane during blunt dissection, use of multiple imaging planes is vital for safe lead positioning. With further evidence of successful implantation, these devices could extend their indications to include patients currently excluded from clinical trials, providing alterative solutions to challenging cases.

## Lead author biography


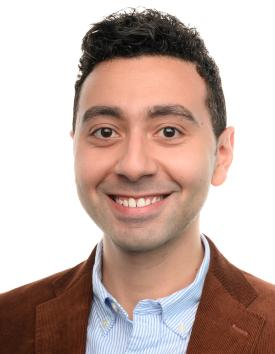


Dr Hesham Aggour is a cardiology specialist registrar at Oxford University Hospitals with a clinical and academic focus in cardiac electrophysiology and implantable devices. He is undertaking a PhD at Imperial College London, where his research explores the implementation of artificial intelligence to enhance cardiovascular risk prediction using ECG data.

**Consent:** The authors confirm that written consent for submission and publication of the clinical case (including images) was obtained from the patient in line with the COPE guidance.

**Funding:** The authors received no financial support for the publication of this article.

## Data Availability

All data are incorporated into the article and its online supplementary material.
